# Mitochondrial genome deletions and minicircles are common in lice (Insecta: Phthiraptera)

**DOI:** 10.1186/1471-2164-12-394

**Published:** 2011-08-04

**Authors:** Stephen L Cameron, Kazunori Yoshizawa, Atsushi Mizukoshi, Michael F Whiting, Kevin P Johnson

**Affiliations:** 1 Discipline of Biogeosciences, Faculty of Science & Technology, Queensland University of Technology, GPO Box 2434, Brisbane, QLD 4001, Australia; 2 Australian National Insect Collection & CSIRO Ecosystem Sciences, Black Mountain Laboratories, PO Box 1700, Canberra, ACT 2601, Australia; 3 Systematic Entomology, Graduate School of Agriculture, Hokkaido University, Sapporo, 060-8589, Japan; 4 Department of Biology, Brigham Young University, Provo, UT, 84602, USA; 5 Illinois Natural History Survey, University of Illinois, 1816 South Oak St., Champaign, IL, 61820, USA

## Abstract

**Background:**

The gene composition, gene order and structure of the mitochondrial genome are remarkably stable across bilaterian animals. Lice (Insecta: Phthiraptera) are a major exception to this genomic stability in that the canonical single chromosome with 37 genes found in almost all other bilaterians has been lost in multiple lineages in favour of multiple, minicircular chromosomes with less than 37 genes on each chromosome.

**Results:**

Minicircular mt genomes are found in six of the ten louse species examined to date and three types of minicircles were identified: heteroplasmic minicircles which coexist with full sized mt genomes (type 1); multigene chromosomes with short, simple control regions, we infer that the genome consists of several such chromosomes (type 2); and multiple, single to three gene chromosomes with large, complex control regions (type 3). Mapping minicircle types onto a phylogenetic tree of lice fails to show a pattern of their occurrence consistent with an evolutionary series of minicircle types. Analysis of the nuclear-encoded, mitochondrially-targetted genes inferred from the body louse, *Pediculus*, suggests that the loss of mitochondrial single-stranded binding protein (mtSSB) may be responsible for the presence of minicircles in at least species with the most derived type 3 minicircles (*Pediculus, Damalinia*).

**Conclusions:**

Minicircular mt genomes are common in lice and appear to have arisen multiple times within the group. Life history adaptive explanations which attribute minicircular mt genomes in lice to the adoption of blood-feeding in the Anoplura are not supported by this expanded data set as minicircles are found in multiple non-blood feeding louse groups but are not found in the blood-feeding genus *Heterodoxus*. In contrast, a mechanist explanation based on the loss of mtSSB suggests that minicircles may be selectively favoured due to the incapacity of the mt replisome to synthesize long replicative products without mtSSB and thus the loss of this gene lead to the formation of minicircles in lice.

## Background

The gene content, order and structure of bilaterian mitochondrial (mt) genomes are amongst the most stable genomic systems known. Gene content within bilaterians, with few exceptions, consists of 37 genes, 13 protein coding genes (PCGs), two ribosomal RNAs (rRNAs) and 22 transfer RNAs (tRNAs). This gene content is stable across 32 phyla, representing 78% of life and 555 million years of evolution. The exceptions such as the loss of the smallest PCG in higher nematodes [1] and of tRNAs in some mites (e.g. [2]), affect only genes which are physically small and these losses are confined to groups representing a small portion of the bilaterian diversity [3]. Gene order is variable but in several instances an ancestral arrangement is shared by the majority of species in enormous groups such as the Superphylum Ecdysozoa (arthropods, velvet worms and priapulids) and for the chordates including most vertebrates [4-7]. Again exceptions such as the highly rearranged mt genomes of lice [8] and wasps [9] within arthropods or those of plethodontid salamanders within vertebrates [10] are comparatively rare and confined to relatively derived portions of each group. Mt genome structure within bilateria is almost universally a single circular genome although it is more variable in lower metazoa and protists [11-13]. This great stability of gene content, arrangement and structure across such an enormous age and diversity of life suggests strong stabilizing selection for an optimal bilaterian mt genome. The few animal groups which diverge from this pattern are thus of particular utility for investigating the potential causes of this near-universal genomic stability.

Recently, two studies have independently reported the most marked departures from this general pattern of mt genomic stability within bilateria found to date: the break up of the canonical single chromosome mt genome into multi-chromosome, minicircular mt genomes. Gibson et al. [14,15] reported that the mt genome of the root parasitic nematode *Globodera *and near relatives was spread over six minicircular chromosomes. Each chromosome had between 2 and 19 genes, with several of the PCGs occurring on multiple chromosomes with evidence for modest nucleotide divergences between these different chromosome copies. Collectively the canonical set of 37 mt genes was present although no single chromosome contained all of the genes. Thus Gibson et al. [14] described the mt genome of *Globodera *to be a mosaic spread across the different chromosomes. A more extreme example was reported by Shao et al. [16] from the human body louse, *Pediculus *in which the mt genome was spread over at least 18 minicircular chromosomes, each containing one to three genes plus a large non-coding region containing sequence blocks that were highly conserved between different chromosomes. Again the canonical 37 mt genes were collectively present across the set of 18 mt chromosomes; however there was no evidence for a full-sized chromosome which contained all 37 genes in *Pediculus *or its close relatives.

The finding of multi-chromosomal, minicircular mt genomes in these groups is even more remarkable when compared against our growing knowledge of mt diseases in humans [17]. A wide range of human diseases have been found to result from the deletion of portions of the mt genome a state termed ΔmtDNA. ΔmtDNA is caused by either mutations within the mt genome itself or defective versions of the nuclear encoded mtgenome maintenance genes [18-21]. The progressive loss of mt function due to ΔmtDNA mutations has also been hypothesized as the ultimate cause of aging: the mitochondrial theory of aging [22,23]. However, whether these mutations are sufficiently severe to cause the range of aging effects has been questioned [24]. Models of human mt disorders have been identified from screens of *Drosophila *mutants lacking or differing in the same replication and nucleotide transport proteins [25], highlighting the fact that these systems have been maintained over large evolutionary distances and are likely shared across bilaterians. The replacement of the canonical mt genome structure in *Pediculus *and *Globodera *with a putatively "pathological" structure as their standard suggests a major departure from these common mt genome maintenance pathways. Accordingly, further investigation of mt genome evolution within these groups could well shed light on the forces shaping stabilizing selection on animal mt genome structure in general.

The mt genomes of lice (Insecta: Psocodea: Phthiraptera) are known to be highly rearranged [8,26,27] and the finding of multi-chromosomal, minicircular mt genomes within *Pediculus *thus must be considered within this context. Recent phylogenetic analyses of lice suggest that the sucking lice (Suborder Anoplura), of which *Pediculus *is a representative, may be derived from within the largest of the three chewing louse suborders, the Ischnocera [28-31]. To date mt genomes are available for just two ischnoceran lice, *Campanulotes *[27] and *Bothriometopus *[8] and neither study reports evidence for multi-chromosomal or minicircular genomes. This study thus had three aims:

1) conduct a broader survey of ischnoceran lice for the presence of minicircular mt genomes;

2) compare genome structure to the evolutionary history of lice; and,

3) mine the recently published nuclear genome of *Pediculus *for nuclear-encoded, mitochondrially-targeted (ne-mt) genes to test if intrinsic factors in their genetic background might be responsible for the minicircularisation of louse mt genomes.

## Results

### Mini-circles are common in lice

**Figure 1:**
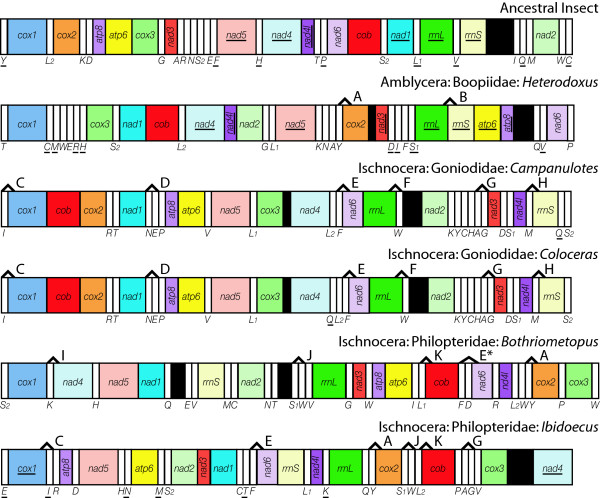
**Gene maps of complete louse mt genomes**. Circular genomes have been arbitrarily linearised on the tRNA gene immediately upstream of *cox1 *to allow ease of comparison. tRNA genes are designated by the single letter amino acid code. Genes which are underlined are encoded on the opposite strand from the majority of genes in that genome. Black gene blocks represent putative control regions. Shared, derived gene boundaries are labeled above each genome; A: *trnY-cox2*; B: *rnsL-rnsS*; C: *trnI-cox1*; D: *trnN-trnE*; E: *trnF-nad6*; E*: *trnF-trnX-nad6*; F: *rnsL-trnW*; G: *trnA-trnG*; H: *trnM-rrnS*; I: *trnK-nad4*; J: *trnS-trnW*; K: *trnL-cob*.

**Figure 2:**
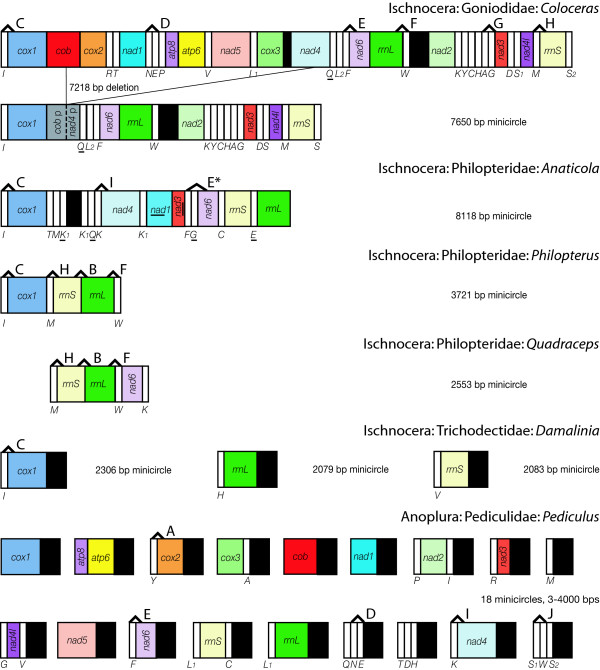
**Gene maps of louse minicirclular mt genomes**. Shared derived gene boundaries are labeled as in Figure 1.

Mt genomes from representatives of six ischnoceran genera were sequenced for this study representing the three most widely accepted ischnoceran families: Philopteridae (*Ibidoecus, Anaticola, Philopterus *and *Quadraceps*), Goniodidae (*Coloceras*) and Trichodectidae (*Damalinia*). Complete mt genome sequences including all protein-coding, rRNA and tRNA genes were determined for *Ibidoecus *(14,908 bp, GenBank accession number: JN122005) and *Coloceras *(14,868 bp; JN122000) (Figure 1). Multigene, minicircular genomes including only a subset of the canonical 37 mt genes were sequenced from *Anaticola *(8118 bp, 18 full length genes; JN121999), *Philopterus *(3721 bp, six genes; JN122006), *Quadraceps *(2553bp, six genes; JN21998) and *Coloceras *(7649 bp, 22 full and two partial genes; JN122001) in addition to the full sized mt genome found in *Coloceras *(Figure 2). Three minicircular genomes each including only a single protein coding or rRNA gene plus one tRNA gene were found in *Damalinia *(2079, 2083, 2306 bp; JN122002, JN122003, JN122004) (Figure 2). Complete details of the sequencing strategies followed to sequence each mt genome are provided in Additional File 1.

The minicircular genomes found in this study fall into three different categories. The *Coloceras *minicircle is one of two heteroplasmic genome types present in this individual louse and has been generated by the deletion of approximately half the mt genome (7218bp covering seven tRNAs, six full length PCGs and portions of two additional PCGs). Heteroplasmy here is used to describe the existence of multiple genome types, not sequence divergences between homologous genes found on different chromosomes as this was not observed in *Coloceras*. The deletion occurs between two poly-T regions, a stretch of 28 T's from bases 710 to 737 in *cob *and of 19 T's from bases 1256 to 1274 in *nad4*. Similar deletions within protein coding genes were detected from *Pediculus *due to recombination between regions of sequence homology however the recombination sites were not homopolymer stretches [32]. The deletion in the *Coloceras *mt genome was consistently detected by amplifications spanning the deleted region; multiple independent primer pairs producing the same result with the shortest using primers 325 bp upstream and 73 bp downstream respectively of the deletion producing a 398 bp product in place of the expected 7600 bp product. Long poly-T stretches are common in the *Coloceras *mt genome. In addition to the two poly-T stretches associated with the deletion there are 23 other poly-T stretches of 10+ bases occurring in eight of the 13 PCGs, in *trnT *and in the major non-coding region (= putative control region). Such long mononucleotide repeats are uncommon in the coding regions of other insects where they are largely confined to the control region. Given this large number of repeats between which deletions could potentially occur, it is perhaps surprising that only a single minicircle type was found. That this single type was found from multiple different PCR primer combinations with varying conditions also argues against it being a PCR artifact caused by polymerase slippage between poly-T regions.

A second minicircle type is found in *Anaticola, Philopterus *and *Quadraceps*. In each of these species we were unable to find a full sized mt genome which included all 37 genes. Unlike in the *Coloceras *minicircle, all genes found on these genomes are full length and there are no mononucleotide stretches of more than eight bases. *Anaticola *and *Philopterus *each have a large non-coding region, 232 and 138 bp respectively, which may function as a control region however the identification of such regions in lice has been problematic as they lack the conserved regions found in most other insects. It thus seems likely that these minicircles are not the result of recent, in evolutionary timescales, deletions from a full sized mt genome as in *Coloceras*. We were able to amplify two additional mt genes from two of these species, *Philopterus *and *Quadraceps*, but unable to link them by long PCR to any of the genes on the minicircles suggesting that the mt genome of these species is now functionally multi-chromosomal. We have, however, not been able to successfully amplify the mt chromosomes on which these genes occur and so we can only infer, rather than demonstrate, a multi-chromosomal mt genome for these species. Multiple primer combinations were used to attempt to amplify the missing genes from each of the species both within individual genes by short PCRs and between genes by long PCRs however we were not able to amplify additional chromosomes from these three species. Next-generation sequencing methods which are not reliant on PCR amplification hold promise in completing these genomes; however, we were not in a position to attempt this as part of the current study. Indeed, next-gen sequencing was the key to completing the sequencing of the multi-partite *Pediculus *mt genome [16] as prior to the nuclear genome sequencing project [33], only two mt genes had been successfully sequenced from this species [27].

**Figure 3:**
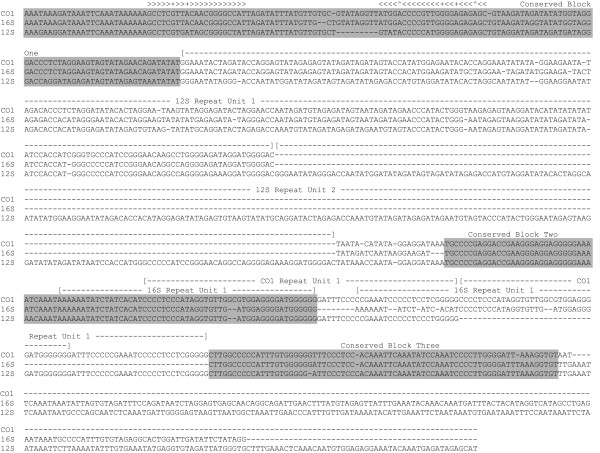
**Alignment of the non-coding regions of three *Damalinia *mt genome chromosomes**. The three conserved blocks are highlighted in gray. Repeat units which differ between the chromosomes are labeled above the alignment with the chromosome on which they are found. The paired bases of the putative stem-loop structure are indicated with a > for 5' side and < for the 3' side of the stem, non-Watson-Crick pairs with a + and bulge bases with a ^.

The third minicircle type was found in *Damalinia *and is very similar to that previously reported from *Pediculus *by Shao et al. [16]. Each of the three minicircles found in *Damalinia *consists of a single PCG or rRNA, a single tRNA and a large non-coding region which displays shared motifs between copies on different minicircles. The non-coding regions are 694bp (*cox1 *containing minicircle), 846bp *(rrnL *containing minicircle) and 1089bp (*rrnS *containing minicircle) long; most of the length differences are due to macrorepeats which vary in number between different chromosomes. The non-coding portion of the genome consists of conserved regions broken up by macrorepeats (Figure 3). The first conserved region, immediately downstream of the coding regions, consists of approx. 150 bp and includes a large hair-pin loop which may function as an origin of replication. This is followed by a less conserved region of 250 bp which is repeated in the *rrnS *minicircle. The third region is another conserved sequence block 142 bp long in the *rrnS *minicircle but longer in the *rrnL *and *cox1 *minicircles due to the presence of two different repeats 53 and 67bp long respectively. The fourth region of 75 bp in the *cox1 *minicircle is also conserved however there are additional highly divergent regions of 171 and 220 bp each in the *rrnL *and *rrnS *minicircles respectively. The overall minicircle structure in *Damalinia*, a small number of genes plus a very large non-coding region composed of regions highly conserved between different minicircles, is very similar to that previously reported for *Pediculus*. If the remainder of the *Damalinia *mt genome has the same structure there could be 12 or more additional minicircles each with a single protein-coding gene and one or more tRNAs.

### Genome Annotations & Evolutionary Trends

Gene order maps for the six ischnoceran species in this study are included in Figures 1 &2. Gene order within the full sized *Coloceras *mt genome is almost identical to that of *Campanulotes *in that they differ by a single tRNA translocation of *trnQ*; these are the first two louse genera to be examined which share more than a handful of plesiomorphic gene boundaries that are common across all insect orders. Additionally, *Coloceras *and *Campanulotes *share the derived arrangement *trnN-trnE *with *Pediculus *but no other louse genera. *Ibidoecus *shares none of the plesiomorphic gene boundaries found in other insects and lacks even the four identified in other lice: *atp8-atp6 *found in *Heterodoxus, Campanulotes, Coloceras *and *Bothriometopus*; *trnG*-*nad3 *found in *Campanulotes, Coloceras *and *Bothriometopus*; *nad4-trnH-nad5 *found in *Bothriometopus*; and *nad4L-nad4 *found in *Heterodoxus*. In contrast *Ibidoecus *shares several derived gene boundaries with other lice: *trnY-cox2 *is shared with *Heterodoxus, Bothriometopus *and *Pediculus*; *trnA-trnG *are shared with *Campanulotes *and *Coloceras*; *trnF-nad6 *is shared with *Campanulotes, Coloceras *and *Pediculus *and with slight modification with *Bothriometopus *(*trnF-trnD-nad6*) and *Anaticola *(*trnF-trnG-nad6*); *trnS-trnW *is shared with *Bothriometopus *and *Pediculus*; *trnL-cob *are shared with *Bothriometopus *and *trnI-cox1 *is shared with all ischnoceran lice except *Bothriometopus*.

The number of missing genes creates some uncertainty about the rearrangement patterns within the partial, minicircular mt genomes. However, none of the four species included here possess any of the plesiomorphic gene boundaries found across insects. Three of these species, *Anaticola, Philopterus*, and *Damalinia*, share the widespread derived gene boundary *trnI-cox1*. *Anaticola *shares the derived boundary *trnK-nad4 *with *Bothriometopus *and *Pediculus *although it has three copies of *trnK *and it is unclear which of these copies is homologous and which are duplicates. *Philopterus *shares *trnM-rrnS *and *rrnL-trnW *with *Campanulotes, Coloceras *and *Quadraceps *and *rrnS-rrnL *with *Quadraceps *and *Heterodoxus*. This rearrangement is, however, sufficiently similar to the insect plesiomorphic arrangement *rrnS-trnV-rrnL *as to be conceivably the product of convergent translocations of *trnV *out of this position occurring independently in each lineage.

**Figure 4:**
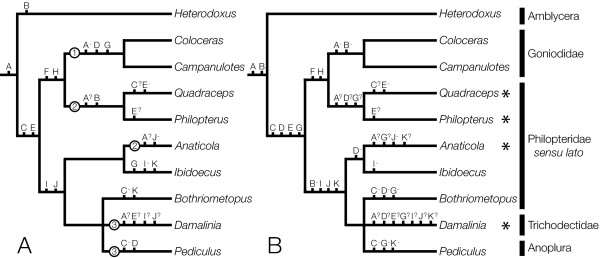
**Mapping genome rearrangements and reductions onto an evolutionary tree of the lice**. Tree is pruned to include just the ten genera for which mtgenome data is available (full tree provided in Additional File 2). A: Derived gene boundaries mapped parsimoniously; B: Boundaries mapped for a single origin and multiple losses. Derived character states are labeled; A: *trnY-cox2*; B: *rnsL-rnsS*; C: *trnI-cox1*; D: *trnN-trnE*; E: *trnF-nad6*/*trnF-trnX-nad6*; F: *rnsL-trnW*; G: *trnA-trnG*; H: *trnM-rrnS*; I: *trnK-nad4*; J: *trnS-trnW*; K: *trnL-cob*. Characters marked with a "-" are inferred secondary losses, characters marked with "?" are unknown states due to partial mtgenomes. Minicirclular mtgenomes types are indicated, 1: heteroplasmic genome reduction; 2: multi-gene minicircles without conserved sequence blocks; 3: single-gene, minicircles with conserved sequence blocks. * indicates partially sequenced genomes.

Shared, derived gene boundaries were mapped onto a phylogenetic tree of the louse genera for which mtgenome data is available, either parsimoniously (Figure 4A), i.e. minimum number of inferred gene translocations even if that infers multiple independent origins of a given gene boundary, or with a single origin for each derived boundary and multiple losses (Figure 4B) (Figure 4 is a pruned copy of the tree in Additional file 2, and is reduced to just taxa for which mt genome data is available). The very closely related goniodid genera *Coloceras *and *Campanulotes *which have almost identical mt genome arrangements share 34 derived boundaries, however 27 of these are not present in any other genus and aren't considered further. There are 11 derived gene boundaries which are shared by at least two louse genera or the goniodids plus at least one other louse (marked A - K, Figures 1, 2 &4). Two of these, *rrnl-trnW *(derived gene boundary F) and *trnM-rrnS *(boundary H) are synapomorphic for the clade ((*Coloceras *+ *Campanulotes*) + (*Quadraceps *+ *Philopterus*)) and are present in all members of that clade without secondary modification or loss. Five derived boundaries (A, C, E, I, J) are interpreted as synapomorphic for various clades under both character mapping schemes, but have been secondarily lost or are unknown in some members of that clade because the partial genomes available for some genera lack both genes. Another four derived boundaries (B, D, G, K) are most parsimoniously interpreted as having evolved convergently in two different genera or clades. However, a single origin optimization for these boundaries requires respectively, one loss and two unknown states (K), or two independent losses (B, D and G, with zero, two and three unknown states respectively).

Mapping genome reductions onto the same tree (Figure 4B) suggests that there is limited evolutionary pattern to the presence of mtgenome minicircles in different louse groups. Type 1 minicircles, heteroplasmic genome reductions from a full sized mtgenome, are confined to the Goniodidae. Type 2 minicircular mtgenomes were found in two different lineages, *Quadraceps*+*Philopterus *and *Anaticola*. Due to the polytomy between *Bothriometopus, Damalinia *and *Pediculus*, type 3 multichromosomal mtgenomes were not inferred to have a single evolutionary origin despite previous louse phylogenies strongly supporting the monophyly of *Damalinia*+*Pediculus *[31] which would suggest a single origin for type 3 minicircles.

### Nuclear Encoded - Mitochondria Targeted Genes of Lice

We initially conducted an annotation of the *Pediculus humanus *ne-mt genes for the Human Body Louse Genome consortium [33] but a fuller discussion of these results follows here. The set of nuclear-encoded, mitochondrially targeted (ne-mt) genes identified from *Drosophila *consists of 315 genes. Reciprocal best blast matches were identified in the *Pediculus *nuclear genome for 295 (93.6%) of these genes with 68 of the 74 (91.9%) oxydative phosphorylation (OXPHOS) genes, 86/87 (98.9%) other metabolic genes, 108/113 (95.6%) protein synthesis genes, 12/15 (80%) DNA synthesis genes, 19/22 (86.4%) transport and cell rescue genes and 2/4 (50%) of the miscellaneous function genes. A table of the annotated genes, their blast and alignment similarity statistics are included in Additional File 3. The numbers of OXPHOS [34-37] and protein synthesis homologues [38] identified from *Pediculus *are comparable to those identified in other insect genome projects. These two subsets are the best studied of the ne-mt genes and represent 59.4% of the mitochondrial gene set. In contrast the DNA synthesis set of *Pediculus *apparently lacks mitochondrial single stranded binding protein (mtSSB), a core member of the mtDNA replisome and has a reduced set of mitochondrial transcription termination factors with only two of the four genes found in *Drosophila*.

Studies of human mt diseases have demonstrated that two classes of disorders will result in mt genome deletions causing minicircles similar to those found in *Pediculus *and *Damalinia*: 1) defects in nucleotide translocator proteins; and 2) defects in any of the mtDNA replicative enzymes [39-41]. Defects in the nucleotide translocator proteins cause imbalances in the intramitochondrial nucleotide pool and so result in a reduction in the rate and accuracy of mtDNA replication. Each of the three nucleotide translocator genes implicated in mtDNA deletions, ADP/ATP carrier protein (= adenine nucleotide translocator 1), deoxynucleoside kinase and ADP-forming succinyl-CoA synthetase, had homologues in *Pediculus *with very high to almost perfect levels of amino acid conservation across insects. There is thus no reason to suppose that the nucleotide translocators are functioning abnormally in *Pediculus*, suggesting that nucleotide imbalances are not the cause of mtDNA minicircles. Of the four core enzymes responsible for mtDNA replication, the replicative helicase Twinkle and DNA polymerase γA (POLGA) are similar to homologues in *Drosophila*, DNA polymerase γB (POLGB) is quite divergent and mitochondrial-specific single-stranded binding protein (mtSSB) is apparently absent. Twinkle in *Pediculus *is moderately divergent from that found in *Drosophila *with limited amino-acid identity through the N-terminal primase domain; of the six conserved motifs only motifs 1 (zinc finger motif), 4 (β-sheet 10, α-helix 3), 5 (β12, α6), and 6 (β13, α7) are conserved. There is much greater similarity in the central linker and C-terminal helicase domains; all of the five conserved motifs in the helicase domain are extremely conserved with the majority of residues identical and most of the remaining ones structurally similar (the structural model of Twinkle follows [19] and [42]). POLGA in *Pediculus *has a similar structure as in other metazoans; the N-terminal exonuclease (exo) domain has moderate similarity to other insects; the C-terminal polymerase (pol) and central linker domains are highly conserved and each of three active sites in both the exo and pol domains are highly conserved across insects (model following [43]). In contrast POLGB in *Pediculus *is highly divergent from that of other insects, lacking much of the N-terminal domain including the helix-loop-helix and leucine-zipper motifs, the central M-domain has limited similar to other insects however the C-terminal domain is highly conserved (model following [44]). This suggests that a minimal mt replisome consisting of a DNA-polymerase (POLG holoenzyme) plus a helicase (Twinkle) is present in *Pediculus*, however, the absence of mtSSB is unique amongst sequenced insects.

## Discussion

### Mt genome rearrangements in Lice

As in all previous studies of lice [8,26,27] all of the mt genomes sequenced for the present study were highly rearranged relative to the inferred ancestral mt genome of insects. Mt genomes from different louse species are also highly rearranged relative to each other. One hundred and seventy two derived gene boundaries are found in the six complete and four minicircular mt genomes sequenced from lice to date (see Additional file 4 for complete list), dwarfing the 12 plesiomorphic gene boundaries which have been retained from the ancestral insect. This pattern is also reflected in every louse species examined where the numbers of derived boundaries is high compared to the number of plesiomorphic boundaries which range from a maximum of five plesiomorphic boundaries in *Bothriometopus *down to a minimum of none in *Ibidoecus*. This confirms earlier claims that lice possess the most rearranged mt genomes amongst the arthropods and are amongst the most rearranged of all animals [26]. The 172 derived gene boundaries are made up of 161 unique gene boundaries found only in a single species, plus 11 shared derived boundaries which occur a total of 31 times across the examined louse species. These ratios are comparable to those found for rearrangements within Hymenoptera where of 67 rearrangements found in the order, 60 were unique to a single wasp species [9].

The present study is the first to find derived gene arrangements that are shared between distantly related louse species. The conserved rearrangements between the two goniodid genera, *Coloceras *and *Campanulotes *confirms the finding of Covacin et al. [27], based on partial genome sequences, that derived gene arrangements are shared at shallow phylogenetic levels within lice. More interestingly, we find the first evidence for shared, derived rearrangements which delimit much higher level groups within the lice e.g. *trnI-cox1 *which appears to be synapomorphic for the Ischnocera although it has been lost in *Bothriometopus *and in the Anoplura; or *rrnL*-*trnW *and *trnM-rrnS *which define a clade consisting of the Goniodidae + (*Quadraceps*+*Philopterus*). Our finding of synapomorphic gene rearrangements within lice is in line with data from other insect orders including Hymenoptera [9,45], Neuroptera [46], Lepidoptera [47], Orthoptera [48,49] and Hemiptera [50], suggesting that rearrangements, when present, are often synapomorphic for major groups within each order. Lice however differ from all other insect orders in the almost complete absence of plesiomorphic gene boundaries as noted above. In contrast, all of the other insect orders cited above except Hymenoptera have only a single derived rearrangement with the remainder of the genome sharing the arrangement of the ancestral insect. Even the most rearranged hymenopteran mt genomes, those of *Bombus *and *Apis*, have only seven derived rearrangements with the rest of the genome in the ancestral arrangement [9,51].

### Minicircles in Lice

The present study shows that mitochondrial minicircles are common in lice being found in six of the ten louse species which have been studied to date. Furthermore, they are evolutionarily widespread within lice with representatives of all three ischonceran families plus the Anoplura possessing minicircular mt genomes. Minicircles have yet to be reported from the other major louse suborder, Amblycera. However little effort has yet been directed towards this group with just one genome sequenced (*Heterodoxus*) versus nine from Ischnocera. We have also identified two additional types of minicircles that could represent intermediate steps between the canonical single chromosome mt genomes found in almost all bilaterians and the small, multichromosomal mt genomes found in sucking lice [16]. While it is tempting to see these two additional minicircles types as an evolutionary series leading to the multichromosomal type 3 minicircle, phylogenetic mapping of the minicircle types simply doesn't support such an interpretation (Figure 4a). The three minicircle types do not form a nested series within the tree and the plesiomorphic, full sized genome is found throughout the tree rather than being confined to some paraphyletic assemblage including the outgroup.

The type 1 minicircles, heteroplasmic genome reductions from a full sized mt genome, are confined to the Goniodidae but however likely represent the first step towards mt genome size reduction. This minicircle type is similar to the ΔmtDNA state found widely in animals and similar mechanisms of DNA deletion could generate the heteroplasmic mt genome variants seen within the Goniodidae. As noted by Shao et al. [16], it is highly unlikely that the much more derived minicircle type seen in *Pediculus *evolved from a "big bang" which fragmented a full-sized mt genome into 18 minicircular chromosomes in a single event. A first intermediate step resembling the heteroplasmic mt genomes seen in *Coloceras *is much more likely even though it is clear that goniodids are not the ancestors of all other ischnoceran lice. Such heteroplasmic genome reductions, however, could not lead to the other minicircles types described here unless multiple independent deletions from the full sized mt genome resulted in a set of mt chromosomes which collectively covered all 37 mt genes, similar to the set of mt chromosomes found in *Globodera *[14,15]. Our available data suggests that there is just one ΔmtDNA chromosome within the *Coloceras *specimens examined. It is also unknown whether the smaller mt genome type found in *Coloceras *is part of the germ line or if the deletion has occurred within the life span of the louse. Examination of additional specimens could shed light on how widely this particular minicircle is found within this species. Human, nematode and *Drosophila *examples of ΔmtDNA, while not germline, are however, generated in a consistent fashion by deletions between repetitive regions [52-55], suggesting that testing between germline and age-related mt degeneration in goniodids will require targeted sequencing of eggs or early instar nymphs. In addition, further study is required to verify if louse species from which full sized genomes have been reported such as *Bothriometopus *and *Ibidoecus *do not also possess type 1 minicircles and if so what the relative balance of genomes types are within different species. The mt genome of *Bothriometopus *possesses multiple repetitive elements including both duplicated tRNAs and non-coding regions which could delimit deletion regions [8].

Type 2 minicircles, genomes with several full length major genes (PCGs or rRNA) but no evidence of a full sized genome, would form the logical missing link between the heteroplasmic type 1 minicircles and the fully reduced type 3 minicircles. The type 2 minicircles that we found most closely resembled the minicircles reported from the nematode *Globodera *[14,15]; however, they differ in several respects. Unlike in *Globodera *we were unable to demonstrate multiple chromosomes. However, our failure to link other mt genes to the finished minicircles suggests the presence of other chromosomes. We also did not find evidence for a mosaic genome with the same gene being found on multiple chromosomes as slight sequence variants as was found in *Globodera*. Type 2 minicircles were also the most widespread minicircle type found in lice occurring in two different clades within the Ischnocera (Figure 4a) which would explain the difficulties which we have encountered trying to amplify louse mt genomes via the traditional long PCR approach. It is our prediction that further surveys will find type 2 minicircles to occur widely within the Ischnocera. However, experimental efforts must be made in each instance to exclude the possibility of apparently type 2 minicircles actually being type 1 minicircles i.e. that a full size mt genome is also present.

The type 3 minicircles (multichromosomal mt genomes with one major gene and a large, conserved non-coding region on each chromosome) are confined to the Anoplura and the Trichodectidae, which are now strongly supported on both morphological and molecular grounds to form a sister-group [31]. The difference between type 2 and type 3 minicircles is largely one of scale: several major genes are present in type 2 but not more than one major gene in type 3 minicircles; the number of tRNAs present in each type is variable but usually more are present in type 2 minicircles (three+ in each studied type 2 minicircle, not more than three in type 3 minicircles); and short non-coding regions without conserved sequence blocks in type 2 versus longer non-coding regions with several conserved sequence blocks in type 3. Additional studies may reveal that type 2 and type 3 minicircles are simply arbitrary points on a continuum of reduced mt chromosome sizes, but given available data, it is convenient to treat them separately at this time. The high degree of sequence conservation within the non-coding regions of each species is likely due to concerted evolution mediated by intramitochondrial recombination [56], and indeed Shao & Barker [32] report chimeric mitochondrial genomes in *Pediculus *composed of the fusion of two minicircles at the non-coding regions. The conserved sequence blocks mediate homologous recombination between different mitochondrial chromosomes [32]. The non-coding regions of *Pediculus *and *Damalinia *mt minicircles show a high degree of structural similarity. There are moderate differences in the sequence of the non-coding region cloned from different chromosomes in each species, however, the entire non-coding region is readily alignable within each species. In both species, the non-coding region has three conserved sequence blocks located at the beginning, middle, and end of the non-coding region. There are also differences between non-coding regions from the two species. Unlike *Pediculus*, the non-coding regions of *Damalinia *mt minicircles are much smaller, ranging in length from 694 to 1098 bp vs 1643 to 2050 bp long in *Pediculus*. The location of a putative stem-loop structure differs in being located within conserved block 1 of *Damalinia *as contrasted to conserved block 2 of *Pediculus*. *Pediculus *lacks the macro-repeat regions that account for much of the variability between the non-conserved regions from different *Damalinia *chromosomes. Finally, there is very limited sequence conservation between the two species, even between the corresponding conserved regions. The other remarkable characteristic of the non-coding regions of these two species is that they are substantially larger than any of the non-coding regions reported from other louse mt genomes. These range in size from 48 bp in *Coloceras *to 284bp in *Bothriometopus*, whereas the smallest non-coding region in *Damalinia *is 694 bp from the *trnI-cox1 *chromosome and in *Pediculus *is 1643 bp for one of the *nad5 *chromosomes. The non-coding regions of *Damalinia *and *Pediculus *mt genomes are much closer in size to those found in many other insect orders which retain both the insect ancestral genome arrangements and the canonical single mt chromosome structure. The nine clones of non-coding regions from *Pediculus *minicircles that were fully sequenced are all larger than the coding regions on their respective chromosomes [16]. While the size of the non-coding region has not been determined for all 18 mt chromosomes identified in *Pediculus*, the sequence conservation between non-coding regions suggests that they will all be 1600bp plus in size and potentially many times larger than many of the coding regions, which were as small as 65bp for one chromosome that contains only a single tRNA gene. It seems counter intuitive that whatever evolutionary forces may have been driving lice towards smaller, minicircular mt genomes has also driven a large expansion in the size of the non-coding regions. Many additional mt genomes from this group will need to be investigated to determine if there truly is a trend toward size increases in the non-coding regions of lice with type 3 minicircular mt genomes and what, if any, function these bloated non-coding regions serve in genome maintenance, replication, or transcription.

### Factors Leading to Mitochondrial Minicircles in Lice

Given that minicircularized mitochondrial chromosomes are very rare across the diversity of life, it is interesting to consider what factors may have given rise to their evolution. Shao et al. [16] proposed that the evolution of minicircular mt genomes coevolved with blood-feeding in the sucking lice (Anoplura). However this hypothesis is contradicted by the available mt genome data. Minicircular mt genomes within lice are not confined to blood-feeding taxa being also found in four of the seven philopterid genera studied which are all feather-feeding [57] and in the trichodectid *Damalinia *which is skin-feeding [58]. Furthermore, minicircular mt genomes are not found in other blood-feeding lice such as the boopiid *Heterodoxus *[26], or in other blood-feeding arthropods such as mosquitoes [59], reduviid bugs [60], or ticks [61], or in other blood-feeding animals such as leeches (Wu et al. *unpublished data*). Within insects, minicircular mt genomes are confined to lice and the specific minicircular mt genome type documented in [16] is confined to a clade minimally consisting of Anoplura + Trichodectidae [31]. Rather than an extrinsic, life-history related explanation relating minicircles in lice to their parasitism, an explanation based on a genetic factor, loss of mtSSB, seems more plausible.

Of the subset of genes which have been related to mt genome deletions, annotation of the *Pediculus *genome found that the only one that was absent, or highly divergent from homologues in other insects, was mtSSB. While it is possible that mtSSB is present in *Pediculus *but so highly modified that our BLAST approach to gene identification did not detect it, the high degree of conservation of this gene in other insects (Additional File 5) suggests that even if it were present it is so highly modified in *Pediculus *as to be non-functional in which case an argument based on its absence is still valid (c.f. [62]).

Functionally mtSSB has five major roles in the upkeep of mt genomes:

1. Binding to single-stranded mtDNA during replication, protecting it from mutation [63,64].

2. Preventing DNA-slippage at stalled replication forks [65].

3. Increasing the processivity of the replisome thus increasing the rate of DNA synthesis [66,67].

4. Stabilizing the triple-stranded D-loop replicative intermediate in vertebrates [68].

5. A major component of mitochondrial nucleoids [64,69,70].

All single-stranded binding proteins have a function in protecting DNA during the single stranded phase of replication [63] and the absence of mtSSB may be responsible for heightened rates of substitution observed within lice [71,72]. The second and third of these functions suggest that in the absence of mtSSB, formation of minicircles would be favored and possibly even evolutionarily adaptive. Viguera et al. [65] have demonstrated that re-initiation of stalled replication forks requires dissociation of the polymerase from the template. In such circumstances, the single-stranded nascent DNA strand can reassociate with other regions of the template strand resulting in replication slippage as the intervening region between the original and new template sites is not replicated. SSBs stabilize the nascent strand while the polymerases are detached and prevent the illicit pairings which result in slippage in a dose dependent manner. That is, low concentrations of SSB result in a mixed population of full length and partial genomes whereas high concentrations can prevent the formation of slipped genome altogether [65]. This model of slippage depends on the presence of repetitive DNA stretches such that the nascent strand could anneal to both locations. This is enhanced by the formation of hairpin-loop secondary structures that bring the repetitive regions into close physical proximity and can cause replication to stall within the repeat region. Just such a deletion between repetitive elements was identified in the *Coloceras *mt genome resulting in the heteroplasmic mix of full length and minicircular mt genomes.

The absence of mtSSB would also have favoured the retention of minicircles once formed. mtSSB stabilizes the single-stranded conformation of the lagging replication strand which increases the rate of replication (processivity of the replisome) and the maximum size of templates which can be replicated. Korhonen et al. [66] have demonstrated that mtSSB increases the size of replication products in a dose dependent manner from around 2000 bp in its absence to over 15000 bp at high concentrations. In the absence of mtSSB complete replication of a full sized mt genome may be impossible. However, the minicircle sized genomes might be sufficiently small to be replicated by polymerase and helicase alone. The minicircles found in *Pediculus *and *Damalinia *are 3-4 and 2-2.5 kb in size respectively, comparable to the replicable sizes found by experimental knock out of mtSSB. It is thus possible to propose that loss of mtSSB would favour both the formation of minicircles through replicative slippage and their intergenerational retention due to an impaired ability to replicate larger mt genomes.

The effect of loss of mtSSB on the final two roles - D-loop stabilization and nucleoid formation - are difficult to assess owing to a complete lack of research on these phenomena in insect models. In mammals mtSSB stabilizes D-loops by reducing the capacity of transcription factor A (TFAM) to remove the RNA priming region and resolve triple-stranded, DNA/RNA hybrid D-loops into double-stranded non-replicative DNA [68]. D-loops have not been directly demonstrated within insects [73]; however, the conserved sequence box (CSB) regions which are critical to D-loop formation in mammals have [74,75]. CSB regions are binding sites for transcription termination factors and the premature termination of transcription at these sites generates the short RNA primers that form the D-loop [76]. It is unknown what, if any, interactions between mtSSB and TFAM are responsible for D-loop persistence in insects and accordingly the impact of the loss of mtSSB on replication initiation cannot be predicted. TFAM has also been shown in mammals to control mtDNA copy number, possibly due to its role in nucleoid formation [77]. TFAM, however, can be excluded as a cause of minicircle formation in *Pediculus *as its structure is very similar to that of TFAMs from vertebrates and other insects in that it possesses two conserved high mobility group (HMG) motifs (the third, C-terminal HMG found in *Drosophila *and *Anopheles *is however absent but is probably Diptera specific). Mitochondrial nucleoids are one of the most poorly understood facets of mtDNA maintenance, composed of multiple mtDNA molecules complexed with protein and have been proposed as the units of mt inheritance [78]. Nucleoids have, however, yet to be studied in an insect model. Most available data on nucleoid composition is from yeast or vertebrate model species and the protein complement of the nucleoid varies widely between different species [66,70,79]. mtSSB is one of the few consistently recovered components of the vertebrate mt nucleoid; however, its function within the nucleoid is unknown and accordingly it is unclear what effect its absence would have.

## Conclusion

Genome deletions and minicircles are common in louse evolution, not being confined to any particular louse subgroup. Three types of minicircles, which do not appear to form an evolutionary series, were identified within lice, however Type 3 minicircles (multichromosomal minicircles with few genes) are likely synapomorphic for the group Trichodectidae+Anoplura. Annotation of the ne-mt genes from the *Pediculus *nuclear genome, suggests that mitochondrial function is largely intact, as the majority of genes are present, despite the extreme divergence of its multichromosomal mt genome from the single chromosomal mt genome found in almost all other bilaterian animals. The absence of one ne-mt gene, mtSSB, could explain the fragmentation of the mt genome into multiple chromosomes. This mechanistic explanation for the occurrence of minicircles in lice is more likely than ones based on a blood-feeding life-history (see [16]). The absence of mtSSB may also have favoured the retention of minicircles once formed due to an incapacity of the mt replisome to replicate full sized mt genomes. The effect of lacking mtSSB would have on other mt functions such as D-loop stabilization and nucleoid formation requires additional research.

## Methods

### PCR amplification and sequencing

Collecting records for louse specimens are given in Additional File 1. All specimens were collected into 100% ethanol and stored at -80°C prior to extraction. DNA was extracted using the DNEasy Tissue Kit (Qiagen, Hilden, Germany) from a single louse and a single DNA extract was used for all PCRs in this study. Sequence and location of all amplification primers used in this study are in Additional file 6. Initial PCRs for *Ibidoecus *amplified three intragenic regions: 383 bp of *cox1 *(primers: L6225/H7005 from [80]); 550 bp of *rrnL *(16Sar/16Sbr from [81]); and 350 bp of *rrnS *(12Sai/12Sbi from [81]) amplified using reaction conditions as described in [82]. These initial sequences where used to design specific long PCR primers which where combined in all possible forward and reverse combinations of attempted long PCRs between the three genes (standard long PCR protocol below). Initial PCRs for *Coloceras, Anaticola, Philopterus *and *Quadraceps *consisted of two long PCRs *rrnL *to *rrnS *(CAM10/CAM7 from [27]) and *rrnS *to *rrnL *(GON1/GON2 from [27]). Long PCRs for bird lice were performed using Elongase (Invitrogen, Carlsbad, California) with the following cycling conditions: 92°C for 2 min; 40 cycles of 92°C for 30 sec, 50°C for 30 sec, 68°C for 12 min; and a final extension step of 68° for 20 min. Sequencing was performed using ABI BigDye ver. 3 dye terminator sequencing technology and run on ABI 3770 or ABI 3740 capillary sequencers. Cycle sequencing conditions were 28 cycles of 94°C/10 sec, 50°C/5 sec, 60°C/4 min. Within each long PCR product the full, double stranded sequence was determined by primer walking (primer sequences available from SLC on request). Initial PCRs for *Damalinia *were generated for *cox1 *(L6225/H7005), *rrnL *(16Sar/16Sbr) and *rrnS *(12Sai/12Sbi) using ExTaq (TaKaRa, Otsu, Shiga) with the following cycling conditions: 94°C for 2 min, 40 cycles of 94°C for 30 sec, 45°C for 30 sec, 65°C for 5 min. Long PCRs for *Damalinia *were generated using LA-Taq (TaKaRa, Otsu, Shiga) with the following cycling conditions: 98°C for 2 min, 40 cycles of 98°C for 30 sec, and 68°C for 15 min (combined annealing/extension step). Due to sequence variability, the non-coding regions of *Damalinia *were cloned prior to sequencing using the pGem-T Easy Vector system (Promega, Madison, Wisconsin) following manufacturer protocols. Sequencing of short PCR, long PCR and cloned fragments from *Damalinia *was performed using the CEQ DNA analysis system (Beckman Coulter, Brea, California) following manufacturer protocols.

### Analysis and Annotation

Raw sequence files were proof read and assembled into contigs in Sequencher ver. 4 (GeneCodes Corporation, Ann Arbor, Michigan). Transfer RNA analysis was conducted using tRNAscan-SE [83] with invertebrate mitochondrial codon predictors and a cove score cut off of 1. Reading frames between tRNAs were found in Sequencher and identified using translated BLAST searches (blastx) [84] as implemented at the NCBI website (http://www.ncbi.nlm.nih.gov/). Annotations of ribosomal RNA gene boundaries were performed by comparison with published models of rRNA secondary structure (e.g. [85]). Evolution of genome structure and rearrangements was assessed by comparison with phylogenetic trees inferred by adding sequences for *cox1*, lsu-rRNA and *wingless *to the alignment used in [31] for the five species included in the present study but not included, *Ibidoecus, Bothriometopus, Coloceras, Quadraceps *and *Philopterus*. Trees were inferred using MrBayes ver3.1.1 [86] using two independent runs, each with 4 chains and unlinked data partitions for 10,000,000 generations and trees were sampled every 1000 generations. The first 1000 trees were discarded as burnin following determination of stationarity in the Bayesian analyses using Tracer ver. 1.4 [87]. Substitution models were estimated for each gene partition using AIC as implemented in MrModeltest 2.3 [88]. Genome rearrangements were coded as binary states or unknown for species from which only partial mtgenomes are available. Character evolution was traced using MacClade ver 4.06 [89] treating character states as unordered. The tree has been uploaded to TreeBASE (accession: http://purl.org/phylo/treebase/phylows/study/TB2:S11666)

Annotation of the nuclear-encoded, mitochondria-targeted (ne-mt) genes within the *Pediculus humanus *genome were conducted using genome assembly phumU1, hosted on the VectorBase website [90]. The most up to date list of ne-mt genes identified from *Drosophila *was compiled from [38,91]. *Pediculus *orthologues of the *Drosophila *ne-mt genes were identified by the best bidirection hit (BBH) approach [92]. Amino acid sequences for the *Drosophila melanogaster *copies of each gene were taken from GenBank and blasted against the predicted peptide set for *Pediculus *(Phum_pep_ver1.1 gene build) using blastP [84] as implemented on VectorBase. Best blast matches within the *Pediculus *genome were confirmed by reciprocal blasting of the inferred peptide against the *Drosophila *genome on GenBank. Only reciprocal best blast pairs are considered orthologous genes and treated further within this study.

## Authors' contributions

Conceived this study: SLC & KPJ. Performed the work and statistical analyses: SLC, KY, AM, MFW & KPJ. Wrote the paper: SLC, KY, MFW & KPJ. All authors read and approved the final manuscript.

## Supplementary Material

Additional file 1 **PCR strategy**. The PCR amplification strategy and collection records for the lice sequenced in this study. Click here for file

Additional file 2 **Full phylogenetic tree of lice and relatives**. Full tree generated after [31] which was pruned to produce figure 2. Click here for file

Additional file 3 **ne-mt genes found in *Pediculus***. Complete list of gene homologues and blast statistics for the annotation of ne-mt genes found in *Pediculus*. Click here for file

Additional file 4 **Novel mt gene boundaries in lice**. Complete list of novel mt gene boundaries found in lice. Click here for file

Additional file 5 **mtSSB alignment**. Alignment of the amino acid sequences of mtSSB genes annotated from insect nuclear genomes. Click here for file

Additional file 6 **Primers**. Primer sequences and combinations used in this study. Click here for file
