# Supplementary material for: Mitochondrial genome deletions and minicircles are common in lice (Insecta: Phthiraptera)
Source: BMC Genomics. 2011 Aug 4;12:394. doi: 10.1186/1471-2164-12-394 (PMC3199782; doi:10.1186/1471-2164-12-394)
Supplement: Additional file 1 — PCR strategy. The PCR amplification strategy and collection records for the lice sequenced in this study. [file 1471-2164-12-394-S1.DOC]

**Additional File 1: Specimen Collection and Amplification Strategy**

**Specimen Collection**

Collection records for the lice used in this study are as follows: *Coloceras* sp. ex zebra dove (*Geopelia striata*), Honolulu, Hawaii, USA by Sarah Bush, Aug. 6^th^ 2001; *Ibidoecus bisignatus* (Nitzch) ex white-faced ibis (*Plegadis chihi*), Arcadia Pa., Louisiana, USA by Kevin P. Johnson, Sept. 3^rd^ 2000; *Anaticola crassicornis* (Scopoli) ex chilöe widgeon (*Anas sibilatrix*), Rio Negro, Argentina by Kevin P. Johnson, Apr. 15^th^ 2001 (host voucher KGM257); *Philopterus* sp. ex field sparrow (*Spizella pusilla*), McClain Co., Illinois, USA by Kevin P. Johnson, May 16^th^ 2001; *Quadraceps* sp. ex bronze-winged courser (*Cursorius chalcopterus*), Buipe, Ghana, by Kevin P. Johnson, Mar. 10^th^ 2003; *Damalinia* *sika* ex sika deer (*Cervus nippon yesoensis*), Nakajima, Lake Toyako, Hokkaido, Japan, March 7^th^ 2002, Kazunori Yoshizawa. (voucher ID: KY397).

**PCR Strategy**

For *Ibidoecus* the 8 possible long PCR combinations of forward and reverse primers aiming to link the three partial gene sequences for *cox1*, *rrnL* and *rrnS* yielded two successful amplicons, *rrnL* to *cox1* (IBID3/IBID5, approx. 8000 bp in size) and *cox1* to *rrnL* (IBID6/IBID4, approx. 8000 bp). No PCRs originating in the preliminary *rrnS* sequence were successful and comparison between this sequence and the annotated copy of *rrnS* from the complete genome suggest that the preliminary sequence is a pseudogene. The completed genome is 14,908 bp in size and includes all the standard mitochondrial protein coding, rRNA and tRNA genes.

In *Coloceras* both of the initial long PCR amplicons *rrnL* to *rrnS* (CAM10/CAM7, approx. 3350 bp) and *rrnS* to *rrnL* (GON1/GON2, approx. 5000 bp) were successful for a mini-circular genome 7649 bp in size that included 22 full length genes, 5 protein coding genes, 2 rRNAs and 15 unique tRNA isotypes plus partial copies of *cob* (617 vs 1125 bp in full length copy) and *nad4* (27 vs 1293 bp) with the intervening genes deleted. Subsequently we amplified a partial *cox2* gene (ISC-C2F/ISC-C2R, approx. 280 bp) which we were able to link to *cob* from the 5’ end (COLO13/ISC-C2R, approx. 1300 bp) and to the control region from the 3’ end (COLO19/COLO22, approx. 6300 bp). The full sized genome is 14,868 bp in size and includes a single copy of each of the canonical 37 mitochondrial genes.

In *Anaticola* an initial long PCR *rrnS* to *rrnL* (GON1/GON2, approx. 2000 bp) and a partial *cox1* sequence (L6225/H7005, approx. 380 bp) were generated. These were linked by the amplicon *rrnL* to *cox1* (CAM10/ANAT5, approx. 1500 bp) and extended with the amplicon *cox1* to *nad4* (ANAT6/N4-J-8924, approx. 2000 bp). A circular molecule was completed by the amplicon *nad4* to *rrnS* (ANAT16/ANAT4, approx. 3500 bp) for a minicircular genome 8,118 bp in size and including 18 genes, 5 protein coding genes, 2 rRNA genes and 11 tRNAs representing 9 different isotypes (3 copies of *trnK*, 2 sequence identical inverted repeats either side of a non-coding region, and a third which differs by a 8bp indel). In *Philopterus* both of the initial long PCR amplicons *rrnS* to *rrnL* (GON1/GON2, approx. 1600 bp) and *rrnL* – *rrnS* (CAM10/CAM7, approx. 2700 bp) were successful for a minicircular genome 3,721 bp in size and including 6 genes, 1 protein coding gene (*cox1*), 2 rRNAs and 3 unique tRNAs. Subsequently we amplified partial *cox2* (ISC-C2F/ISC-C2R, approx. 280 bp) and *cob* sequences (ISC-CbF/ISC-CbR, approx. 600 bp) but were unable to link either of these sequences either to each other or to the minicircular genome by long PCRs using specific primers designed from each of these sequences. In *Quadraceps* one of the initial long PCRs *rrnS* to *rrnL* (GON1/GON2, approx. 1500 bp) was successful. This sequence was used to design specific primers for the amplicon *rrnL* to *rrnS* (QUAD3/QUAD4, approx. 1500 bp), completing a minicircular genome 2,553 bp in size and including 6 genes, 1 protein coding gene (*nad6*), 2 rRNAs and 3 unique tRNAs. Subsequently we amplified partial *cox1* (L6625/H7005, approx 380 bp) and *cob* (ISC-CbF/ISC-CbR, approx. 600 bp) but were unable to link either of these sequences either each other or to the minicircular genome by long PCRs using specific primers designed from each of these sequences.

In *Damalinia,* primers designed within the initial, partial sequences of *cox1* (COI.out.DS.F/ COI.out.DS.R), *rrnL* (16S.out.DS.F/ 16S.out.DS.R) and *rrnS* (12S.out.DS.F/ 12S.out.DS.R), failed to amplify any long PCRs linking these genes. These primers were then used to search for mitochondrial minicircles which include each of these genes by using primers at the 5’ and 3’ ends of each of the partial sequences. Each resulted in a small amplicon of approx. 2000 bp which was sequenced after cloning. Attempts to amplify other mt genes based on *cox2* and *cob* primers conserved across Ischnocera failed. Attempts to design “universal” *Damalinia* primers based on portions of the non-coding regions conserved across different minicircles (see Fig. 2) also failed to amplify additional genes or minicircles types.
