# Supplementary material for: Mitochondrial genome deletions and minicircles are common in lice (Insecta: Phthiraptera)
Source: BMC Genomics. 2011 Aug 4;12:394. doi: 10.1186/1471-2164-12-394 (PMC3199782; doi:10.1186/1471-2164-12-394)
Supplement: Additional file 4 — Novel mt gene boundaries in lice. Complete list of novel mt gene boundaries found in lice. [file 1471-2164-12-394-S4.DOC]

Additional File 4. Derived gene boundaries for each genus. Genes marked with a superscript “-” are encoded on the minority strand. Shared derived boundaries are labeled with the same letter codes used on figures 1 and 2.

*Heterodoxus*

1. *trnT*-*cox1*
2. *cox1*-*trnC*^-^
3. *trnC*^-^-*trnM^-^*
4. *trnM^-^*-*trnW*
5. *trnW*-*trnE*
6. *trnE*- *trnR^-^*
7. *trnR*^-^-*trnH^-^*
8. *trnH^-^*-*cox3*
9. *cox3*-*trnS*
10. *trnS*-*nad1*
11. *nad1*-*cytB*
12. *cytB*-*trnL*
13. *trnL*-*nad4*
14. *nad4l*-*nad2*
15. *nad2*-*trnG*
16. *trnG-trnL*
17. *trnL*-*nad5*
18. *nad5*-*trnK*
19. *trnK*-*trnN*
20. *trnN*-*trnA*
21. *trnA*-*trnY*
22. *trnY*-*cox2* **A**
23. *cox2*-*nad3*^-^
24. *nad3*^-^-*trnD*
25. *trnD*-*trnI*
26. *trnI*-*trnF^-^*
27. *trnF^-^*-*trnS*
28. *trnS*-*rrnL*
29. *rrnL*-*rrnS* **B**
30. *rrnS*-*atp6*
31. *atp8*-*trnQ^-^*
32. *trnQ^-^-trnV^-^*
33. *trnV^-^-nad6*
34. *nad6*-*trnP*
35. *trnP*-*trnT*

*Campanulotes*/

*Coloceras* (both)

1. *trnI-cox1* **C**
2. *cox1*-*cytB*
3. *cytB-cox2*
4. *cox2-trnR*
5. *trnR-trnT*
6. *trnT-nad1*
7. *nad1-trnN*
8. *trnN-trnE* **D**
9. *trnE-trnP*
10. *trnP-atp8*
11. *atp6-trnV*
12. *trnV-nad5*
13. *nad5-trnL*
14. *trnL-cox3*
15. *cox3-nad4*
16. *nad4-trnL*
17. *trnL-trnF*
18. *trnF-nad6* **E**
19. *nad6*-*rrnL*
20. *rrnL-trnW* **F**
21. *trnW-nad2*
22. *nad2-trnK*
23. *trnK-trnY*
24. *trnC-trnH*
25. *trnH-trnA*
26. *trnA-trnG* **G**
27. *nad3-trnD*
28. *trnD-trnS*
29. *trnS-nad4l*
30. *nad4l-trnM*
31. *trnM-rrnS* **H**
32. *trnS-trnI*

*Campanulotes* (only)

1. *rrnS-trnQ^-^*
2. *trnQ^—^trnS*

*Coloceras* (only)

1. *nad4-trnQ^-^*
2. *trnQ^-^-trnL*

*Bothriometopus*

1. *trnS-cox1*
2. *cox1-trnK*
3. *trnK-nad4* **I**
4. *nad5-nad1*
5. *nad1-trnQ*
6. *trnQ-trnE*
7. *trnE-trnV*
8. *trnV-rrnS*
9. *rrnS-trnM*
10. *trnM-trnC*
11. *trnC-nad2*
12. *nad2-trnN*
13. *trnN-trnT*
14. *trnT-trnS*
15. *trnS-trnW* **J**
16. *trnW-trnV*
17. *rrnL-trnG*
18. *nad3-trnW*
19. *trnW-atp8*
20. *atp6-trnI*
21. *trnI-trnL*
22. *trnL-cytB* **K**
23. *cytB-trnF*
24. *trnF-trnD*
25. *trnD-nad6*
26. *nad6-trnR*
27. *trnR-nad4l*
28. *nad4l-trnL*
29. *trnL-trnW*
30. *trnW-trnY*
31. *trnY-cox2* **A**
32. *cox2-trnP*
33. *trnP-cox3*
34. *cox3-trnW*
35. *trnW-trnS*

*Ibidoecus*

1. *trnC*^-^-*cox1*^-^
2. *cox1*^-^-*trnI^-^* **C**
3. *trnI^-^-trnR*
4. *trnR-atp8*
5. *atp8-trnD*
6. *trnD-nad5*
7. *nad5-trnH*
8. *trnH-trnN^-^*
9. *trnN^-^-atp6*
10. *atp6-trnM^-^*
11. *trnM^-^-trnS*
12. *trnS-nad2*
13. *nad2-nad3*
14. *nad3-nad1*
15. *nad1-trnC*
16. *trnC-trnT^-^*
17. *trnT^-^-trnF*
18. *trnF-nad6* **E**
19. *nad6-rrnS*
20. *rrnS-trnL*
21. *trnL-nad4l*
22. *nad4l-trnK^-^*
23. *trnK^-^-rrnL*
24. *rrnL-trnQ*
25. *trnQ-trnY*
26. *trnY-cox2* **A**
27. *cox2-trnS*
28. *trnS-trnW* **J**
29. *trnW-trnL*
30. *trnL-cytB* **K**
31. *cytB-trnP*
32. *trnP-trnA*
33. *trnA-trnG* **G**
34. *trnG-trnV*
35. *trnV-cox3*
36. *cox3- nad4*
37. *nad4-trnE^-^*

*Anaticola*

1. *trnI-cox1* **C**
2. *cox1-trnT*
3. *trnT-trnM*
4. *trnM-trnK^-^*
5. *trnK^-^-trnK*
6. *trnK-trnQ^-^*
7. *trnQ^-^*-*trnK*
8. *trnK-nad4* **I**
9. *nad4-nad1*^-^
10. *nad1*^-^-*nad3*^-^
11. *nad3*^-^-*trnF*
12. *trnF*-*trnG^-^*
13. *trnG^-^*-*nad6*
14. *nad6-trnC*
15. *trnC-rrnS*
16. *rrnS-trnE^-^*
17. *trnE^-^*-*rrnL*
18. *rrnL-trnI*

*Philopterus*

1. *trnI-cox1* **C**
2. *cox1-trnM*
3. *trnM-rrnS* **H**
4. *rrnS-rrnL* **B**
5. *rrnL-trnW* **F**
6. *trnW-trnI*

*Quadraceps*

1. *trnM-rrnS* **H**
2. *rrnS-rrnL* **B**
3. *rrnL-trnW* **F**
4. *trnW-nad6*
5. *nad6-trnK*
6. *trnK-trnM*

*Damalinia*

1. *trnI-cox1* **C**
2. *trnH-rrnL*
3. *trnV-rrnS*

*Pediculus*

1. *trnP-nad2* **A**
2. *nad2-trnI*
3. *trnR-nad3*
4. *trnK-nad4* **I**
5. *trnG-nad4l*
6. *nad4l-trnV*
7. *trnF-nad6* **E**
8. *trnL-rrnS*
9. *rrnS-rrnC*
10. *trnL-rrnL*
11. *trnQ-trnN*
12. *trnN-trnE* **D**
13. *trnT-trnD*
14. *trnD-trnH*
15. *trnS-trnW* **J**
16. *trnW-trnS*
