# Supplementary material for: Mitochondrial genome deletions and minicircles are common in lice (Insecta: Phthiraptera)
Source: BMC Genomics. 2011 Aug 4;12:394. doi: 10.1186/1471-2164-12-394 (PMC3199782; doi:10.1186/1471-2164-12-394)
Supplement: Additional file 5 — mtSSB alignment. Alignment of the amino acid sequences of mtSSB genes annotated from insect nuclear genomes. [file 1471-2164-12-394-S5.DOC]

Additional File 5. mtSSB alignments. Amino acid sequences of mtSSB from published insect genome sequencing projects. Conserved sites are indicated with a * for fixed sites, amino acids are colour-coded by physicochemical property group.

* * ****

Drosophila MQHTRRMLNP L---LTGLRN LPARGATTTT AAAPAKVEKT VNTVTILGRV

Anopheles ---------- ---MLQFSKF LTRTGLHRAY CTDPTRIEKT VNTVTLLGRV

Tribolium MALFKSSTLS SILRCTKNVK ILRPASSTTT DQEPSRVEKS INSVQLLGRV

Bombyx --MLSSIRTP T---LIRVFF GGQRLHTSVA RLEGQKNEKT INQVTLLGRV

Apis ---------- ---------- ---------- ---------- INQVTLLGRV

Nasonia -MMLNKIFVN T---CKQIRG INYVSARAFT ANDSNKIEKS LNQVTLLGRV

Acyrthosiphon -MLKTKILSS ILRSTNYSKQ LFRNNYTQEA APLPIKIEKT INKVTLLGRV

* * * *** * * ** * * * ** * * **

Drosophila GADPQLRGSQ EHPVVTFSVA THTNYKYENG DWAQRTDWHR VVVFKPNLRD

Anopheles GADPQRRGND EHPVVMFSVA THSNYKYESG DWMQKTDWHR VVVFKPGLRD

Tribolium GADPQKKGSE EHPIAVFSLA THTNYRYESG QFMQRTEWHR VICFKPGLRE

Bombyx GADPQKRGSE EHPVINFPLA THYSYKYESG DILQRTDWHR VSIFKPGLRD

Apis GGEAQKKGSN EHPVVIFSLA THNNYKYTNG DIVQRTDWHK ICVFKPNLRE

Nasonia GGEPQKRGNE EHPVVTFSLA THINYKYEGG DLMQKTDWHR IAVFKPNLRE

Acyrthosiphon GADPQKRGTE EHPVVVFSLA THQNYINNNE ESTQKTDWHR IVVFRPGLRD

* *** * * * ** * *** *

Drosophila TVLEYLKKGQ RTMVQGKITY GEITDQQGNQ KTSTSIIADD VLFFRDANN-

Anopheles AVMSYLKKGQ RTMVTGKITY GEITDQEGKQ RGTTSIIADD VIFLQN----

Tribolium TILNYLKKGQ RVHVTGRITY GEITGEDGKP KSTTAIAADD VIFFQSSPQ-

Bombyx TVYKYLKKGQ RIYVTGKLSY GEVKLDDGQV RTASTVMADD VIFFQSTPYE

Apis NVYTYLKKGQ RVLVSGKISY GEYKDEEGAI KSTTAVIADD VIFFHHNN--

Nasonia NVYNYMRKGQ RVMVNGRLSY GEVKDEDGNM RTATSIIADD IIFFQ-----

Acyrthosiphon TVYNYLQKGQ RIHISGRLIY GELKDESGTS RTTTSIAADD IIFFNSKN--

Drosophila ---

Anopheles ---

Tribolium ---

Bombyx SEQ

Apis ---

Nasonia ---

Acyrthosiphon ---
