# Supplementary material for: Mitochondrial genome deletions and minicircles are common in lice (Insecta: Phthiraptera)
Source: BMC Genomics. 2011 Aug 4;12:394. doi: 10.1186/1471-2164-12-394 (PMC3199782; doi:10.1186/1471-2164-12-394)
Supplement: Additional file 6 — Primers. Primer sequences and combinations used in this study. [file 1471-2164-12-394-S6.DOC]

Additional file 6 Primers, sequence and location for PCR amplifications

| Region | Primer Pair  (F & R) | Location*^a^* | Sequence (5’ →3’) |
| --- | --- | --- | --- |
| General |  |  |  |
| *cox1* | L6625^b^ | 701 | CCG GAT CCT TYT GRT TYT TYG GNA AYC C |
|  | H7005^b^ | 1081 | CCG GAT CCA CNA CRT ART ANG TRT CRT G |
| *cox2* | ISC-C2F^c^ | 334 | TGG TCA YCA RTG RTA TTG RTC HTA TG |
|  | ISC-C2R^c^ | 586 | WCC ACA AAT TTC BGA RCA YTG ACC |
| *cytB* | ISC-CbF^c^ | 275 | TYC GDW RDR TTC ATG CTA ATG GWG C |
|  | ISC-CbR^c^ | 880 | AGA AAA HAM YAA WGC YAY YAC MCC TCC |
| *rrnL* | 16Sar^d^ | 538 | CGC CTG TTT AAC AAA AAC AT |
|  | 16Sbr^d^ | 1030 | CCG GTC TGA ACT CAG ATC ACG T |
| *rrnS* | 12Sai^d^ | 304 | AAA CTA GGA TTA GAT ACC CTA TTA T |
|  | 12Sbi^d^ | 645 | AAG AGC GAC GGG CGA TGT GT |
| *rrnL-rrnS* | CAM10^e^ | 854 | AGA GGG ACG AGA AGA CCC TAT AGA TCT TA |
|  | CAM7^e^ | 544 | CTT CTT CAT AAT CTA CAC CTT TAC TTG C |
| *rrnS-rrnL* | GON1^e^ | 171 | AAD WGT TGT GCC AGC WCT AGC GG |
|  | GON2^e^ | 1158 | AGA ATC TGA CCT GAC TYR CGT CGG TC |
| *Ibidoecus* |  |  |  |
| *rrnL-cox1* | IBID3^f^ | 674 | TCT AGA ATG AAT GGT TTG ATG AGG |
|  | IBID5^f^ | 866 | TGG GCA CAT CAT ATG TTT ACT GTA GG |
| *cox1-rrnL* | IBIB6^f^ | 980 | ACC TTC ATA ATG AAG ATA CTG ACC |
|  | IBID4^f^ | 771 | TCG TTC CAC ATT ATC TTG TCG TAC G |
| *Coloceras* |  |  |  |
| *cob-cox1* | COLO13^f^ | 417 | AGC ATT TAT TGG ATA TGT TCT TCC G |
|  | ISC-C2R^c^ | 586 | AGA AAA HAM YAA WGC YAY YAC MCC TCC |
| *cox2-*control reg. | COLO19^f^ | 543 | AAG GTT GAT GCG ATT CCT GGT CGG |
|  | COLO22^f^ | 23 | AGT AAT TAC TAC ACT AAC CTT TCC |
| *Anaticola* |  |  |  |
| *rrnL-cox1* | CAM10^e^ | 854 | AGA GGG ACG AGA AGA CCC TAT AGA TCT TA |
|  | ANAT5^f^ | 736 | ATG ATG RGC TCA MAC WAC AAA YCC |
| *cox1-nad4* | ANAT6^f^ | 938 | AGC AAC TAT AAT TAT TGC TGT TCC AAC AGG |
|  | N4-J-8924^e^ | 580 | GGA GCT TCA ACA TGA GCT TT |
| *nad4-rrnS* | ANAT16^f^ | 383 | ACC AGA GCG AAT TAT AGC TGT TCG G |
|  | ANAT4^f^ | 637 | ACT TAT CCT ATA AGG AGT GAC GGG C |
| *Damalinia* |  |  |  |
| *cox1* non-coding | CO1.out.DS.F^f^ | 1059 | TCT CTT CAC CAT TGG AGG TCT CAC TGG C |
|  | CO1.out.DS.R^f^ | 844 | ATA TGT GRT GAG CTC ACA CTA CAA ACC C |
| *rrnL* non-coding | 16S.out.DS.F^f^ | 983 | TGG TGT TTG TGA CCT CGA TGT TGA ATA G |
|  | 16S.out.DS.R^f^ | 640 | ATT CAT GAC TAT CCT CCA ATC AAG AGG C |
| *rrnS* non-coding | 12S.out.DS.F^f^ | 562 | TAA GTC AGG TAA ATG CTA AGG AAT GCG G |
|  | 12S.out.DS.R^f^ | 403 | TGG TGT GTT GTC CTT TAG TGG GCA TAC G |

*^a^* Location of 3’ base of primer, relative to 5’ end of the gene in which it is found

*^b^* Primers from Hafner et al. (1994)

*^c^* Primers newly designed, from Ischnocera consensus sequences

*^d^* Primers from Simon et al. (1994)

*^e^* Primers from Covacin et al. (2006)

*^f^* Primers newly designed, specific to this louse species
